# Patients’ recommendations to improve help-seeking for vaginismus: a qualitative study

**DOI:** 10.1186/s12905-024-03026-x

**Published:** 2024-03-30

**Authors:** Rashmi Pithavadian, Tinashe Dune, Jane Chalmers

**Affiliations:** 1 School of Health Sciences, PhD Candidate, Western Sydney University, Locked Bag 1797, Penrith, NSW 2751 Australia; 2 Translational Health Research Institute, Western Sydney University, Locked Bag 1797, Penrith, NSW Australia; 3 Allied Health and Human Performance, University of South Australia, Adelaide, SA Australia

**Keywords:** Vaginismus, Help-seeking, Patient perspective, Recommendations, Pelvic pain, Qualitative, Feminist theory

## Abstract

**Background:**

Research to improve healthcare experiences for women with vaginismus tends to be produced from the perspective of healthcare professionals or health-based researchers. There is lacking research on women’s experiences and recommendations to improve help-seeking for vaginismus from their perspective. To address this research gap, this qualitative study aimed to identify the issues that women face when help-seeking for vaginismus and their recommendations to address it. This sought to support the wellbeing of patients to advocate for their healthcare needs which is often overlooked.

**Methods:**

Using a feminist theoretical approach, semi-structured interviews were conducted with 21 participants who sought help for their vaginismus. Thematic analysis was employed to analyse participants’ recommendations.

**Results:**

Four main themes emerged: *Increase awareness of vaginismus*, *Dismantle myths about sex*, *Destigmatise vaginismus*, and *Empower people with vaginismus during medical consultations*. Subthemes were identified as actionable strategies that participants recommended to improve help-seeking and healthcare for vaginismus.

**Conclusions:**

The findings from this study can inform healthcare practice and policy to foster better synchronicity between health professionals and their patients’ perceptions and expectations of treating vaginismus. This can promote more acceptance of patients’ advocacy of their needs and goals to improve the therapeutic alliance and treatment outcomes for vaginismus in healthcare practice. The strategies recommended to increase awareness of vaginismus and challenge its stigma should be considered in policy to incite a culture of change in healthcare practice and broader society.

**Supplementary Information:**

The online version contains supplementary material available at 10.1186/s12905-024-03026-x.

## Background

Women who have vaginismus often face barriers to seek and receive appropriate help and healthcare for the condition. This is because vaginismus is an overlooked yet common female sexual pain disorder [1, 2]. Vaginismus causes the vagina to involuntarily close with any attempt of penetration, whether by a penis, tampons, fingers, or speculum, for at least six months [3]. This leads to vaginal penetration being painful, difficult, and/or impossible depending on the grade of vaginismus [4]. Vaginismus can be lifelong (primary) or develop after a period of experiencing vaginal penetration without issues (secondary) [5]. While the exact incidence of vaginismus is unknown, the condition is estimated to affect anywhere between 1 and 7% of the global female population [2]. Despite recent varied terminology of vaginismus, such as in the Diagnostic and Statistical Manual 5 (DSM-5), this paper will focus on vaginismus because the term vaginismus is still used in clinical settings [1].

The impacts of vaginismus on women can be devastating. Women with vaginismus often feel inadequate, undesirable, and worthless [5]. The condition is a leading cause of unconsummated relationships and marriages [6]. Vaginismus impacts women’s intimacy with their partners and their ability to start a family. Women with vaginismus tend to have higher rates of mental health issues such as depression, anxiety, and suicidal ideation, and exacerbated pre-existing mental health illnesses [7]. The stress to seek help for chronic pelvic pain issues, such as vaginismus, can have wide-ranging impacts on women’s energy levels, sleep, and performance at work or school [8]. This highlights the importance for women to be able to advocate for their access to appropriate healthcare and treatment.

Conventional and conservative treatments for vaginismus include vaginal trainers (or dilators), pelvic floor physiotherapy, Kegel exercises, and psychotherapy [9]. Recently, more invasive treatments, such as Botox and nerve blocks, are being used [9, 10]. Research has shown that completed treatment for vaginismus has a high success rate to relieve women’s symptoms [9, 11]. However, many studies recognise significant barriers that women face [1, 2].

Challenges to help-seeking for vaginismus can lead to reduced rates of treatment success in clinical practice [1, 2]. In health contexts, help-seeking behaviour refers to searching for help or information to gain treatment and/or relief for a presenting health issue [12]. Research indicates that the medical system can be androcentric which has led to women not receiving appropriate support for their sexual health problems [13]. Many healthcare professionals are also lacking understanding of vaginismus as a condition, its symptoms, and its treatment [2, 9]. This makes it more likely for women with vaginismus to receive incorrect diagnoses or treatment, and having their experiences overlooked [9, 14]. Moreover, there is low social awareness of vaginismus, which means that women often do not realise that they have the condition to know to seek help, nor where or how to seek help for it [2].

Research on vaginismus has been clinically focused on the affected genitalia, symptomology, and treatment [15–18]. There is a lack of research on women’s experiences of seeking and receiving help for vaginismus from their perspective [18]. The existing research on the recommendations to improve healthcare experiences for women with vaginismus is largely produced from the perspective of healthcare professionals or health-based researchers [9]. However, people with vaginismus have important knowledges on the lived experiences of the condition and navigating the healthcare system. Their direct feedback and recommendations are therefore pivotal to identify and address issues in healthcare management of vaginismus. Yet, even in research that examines large groups of patients with vaginismus, the focus is on women’s symptoms and behavioural responses to penetration or treatment [15–17]. Women are not explicitly asked to share their personal recommendations to address problems that they face when help-seeking and for treatment management [9].

When people with health conditions, such as vaginismus, are not directly asked for their perspective to improve healthcare for their experiences, their views become marginalised and/or excluded. This results in the dominance of healthcare professionals’ and researchers’ perspectives and opinions to improve women’s experiences with vaginismus. Given the lacking awareness of vaginismus, women with vaginismus are already marginalised and face challenges to seek and receive help for their health. Therefore, it is paramount to centre the marginalised voices of people with vaginismus to understand their help-seeking experiences and suggestions. This will provide insider insight to implement strategies that closely align with the needs of people who have vaginismus. This study aimed to identify the issues that women face when help-seeking for vaginismus and their recommendations to address it. To address this aim, this study sought to answer the following research questions:

1. What do Australian women, who have been diagnosed with vaginismus, identify as being issues that they face when help-seeking for vaginismus?
2. What are these women’s recommendations to address the issues that they identified?

## Methods

### Study design

The data used to inform the findings presented in this study are part of a larger project focused on women’s experiences of help-seeking for vaginismus. A retrospective qualitative research design was used to capture the holistic accounts and multiple realities of participants’ lived experiences in this study [19]. This produced rich and nuanced data focused on the meanings and interpretations that participants constructed towards help-seeking for vaginismus [19]. A qualitative research design helped to give women autonomy to share their diverse individual experiences while recognising their collective or typical experiences [20]. The Standards for Reporting Qualitative Research (SRQR) were followed to report on this qualitative study [21].

### Theory

A feminist theoretical framework was used because it aims to decentralise the exalted views of dominant groups, such as health professionals and broader society, to advocate the subjective experiences of marginalised people such as those with vaginismus [22]. Specifically, the study used a constructivist feminist approach that does not perceive a single objective truth to be researched [23]. Rather, this approach seeks to give marginalised women the autonomy to advocate for their often unheard truths regarding help-seeking for vaginismus. The study’s research questions were developed to align with a feminist constructivist approach to legitimise women as knowers and producers of knowledge regarding their experiences of vaginismus [24, 25]. Given that many of the recommendations regarding help-seeking with vaginismus is from health professionals or healthcare researchers, this paper aimed to use a feminist approach to centre and advocate the recommendations of those with vaginismus to improve help-seeking and healthcare practice. While the term ‘client/s’ is often used to describe those who receive mental healthcare, recipients of broader healthcare are still referred to as patients [26]. The use of the term ‘client/s’ is also often used in non-healthcare settings. Therefore, the word ‘patient’ seemed more appropriate to describe the perspectives of the participants in this study, and their experiences to negotiate their marginalisation to receive healthcare using a feminist approach.

### Interview guide development

The semi-structured interview guide was developed by following Kallio et al.’s [27] guidelines for the larger study that examined women’s help-seeking experiences for vaginismus. The flexibility of semi-structured interviews was deemed appropriate because it aligned with a feminist approach by enabling participants to vocalise and advocate their views, beliefs, and experiences in their own words and diverge from the script to share unexplored knowledges if they wished [27]. The overarching questions and probes of the preliminary interview guide was informed by a review of the literature and knowledge from the second and third authors’ health professional backgrounds in clinical psychology and physiotherapy [27]. The findings from participants’ responses to interview probes on their suggestions and recommendations to improve help-seeking and healthcare for vaginismus are reported in this paper. The preliminary interview guide underwent internal testing with all the authors and then field testing to add and refine the questions. The final demographic questionnaire and interview guide are presented in Supplementary Tables 1 and 2.

### Participant recruitment and procedure

Criterion sampling, as a subset of purposive sampling, was used for recruitment [28]. The eligibility criteria to participate in the study were people who: 1). had received a diagnosis of vaginismus from a health professional; 2) lived in Australia; and 3) were aged 18 years or above. Between January and May 2020, the recruitment information, which included a summary of the project, eligibility criteria, AUD$25 voucher incentive, and contact email, was circulated to attract participants. Recruitment information on flyers were stuck on bulletin boards and in public women’s bathrooms across the different campuses of Western Sydney University. The authors contacted three national diversity organisations, four national sexual health organisations, three physiotherapy clinics in Sydney, and one physiotherapy clinic in Perth to help with recruitment. The authors also circulated the study information on their personal and professional social media accounts on Facebook, Twitter, and LinkedIn. The first author contacted the administrators of four vaginismus support groups on Facebook and moderators of eight sexual health community pages on Reddit to post recruitment information. Two participants of the study also circulated recruitment information through word-of-mouth. Fifty-four people contacted the first author with interest to participate in the study. The participant information sheet and consent forms were emailed to the 54 people. After establishing eligibility, 21 participants attended interviews.

### Data collection

The first author conducted interviews with 21 participants between January and May 2020 via Zoom videoconferencing software. To follow a feminist approach and promote inclusivity of the marginalised participant group, interviews through Zoom were used to overcome spatial boundaries and travel costs to include the voices of people from around Australia [29]. Interviews averaged 1 h and 30 min (range 54 min to 2 h and 54 min). The interviews were audio recorded on Zoom and transcribed verbatim using Trint transcription software. Transcripts were then manually reviewed, and the audio file was validated against the transcription [30]. Idiosyncrasies of speech, such as word repetition, were retained in transcripts to avoid altering participants’ expression. All participants were emailed their respective de-identified transcript for member-checking before data extraction began. The audio-recordings of interviews and transcripts are located in restricted access data storage [31].

### Data analysis

While descriptive statistics were used to analyse the data produced from the demographic questions, inductive thematic analysis was chosen to identify, analyse, and report patterns or themes within the interview data [32]. Thematic analysis was employed as a feminist tool to recognise participants’ marginalised individual experiences while categorising the similarities and differences between them [33]. The 21 transcripts were each read three times for familiarity and to inform the construction of a preliminary coding frame to answer the research questions through a feminist lens to centre participants’ voices.

Quirkos, a computer-assisted qualitative data analysis software, was used as a practical tool to code every line of the transcripts according to the preliminary coding framework. This helped in the process of revising and refining the coding frame by merging codes and identifying new codes [34]. Data and theoretical saturation was reached with 21 interviews as the constructed themes answered the research questions and no new data, themes, nor coding was attainable within the scope of the study [35]. After coding, participants’ recommendations emerged as four themes.

### Disclaimer

This study recognises that gender is a social construct and therefore not all persons who experience vaginismus would identify as a woman. Someone may have vaginismus and a non-binary gender identity or be intersex. Given the scope of this study and nearly all participants identifying as women, it is not feasible for the study to sufficiently consider the collective experiences of non-binary gender individuals’ help-seeking for vaginismus, which would be another area of specialised research. Nevertheless, since one participant identified as agender, inclusive language of non-gendered terms such as ‘they/them/their’, ‘participants’, ‘people’ or ‘patients’ (rather than the word ‘woman/women’) were used to refer to findings that included this participant.

### Researcher reflexivity

All three authors identify as cis-gender women with varied help-seeking experiences in the healthcare system. While such reflexivity aided the feminist constructivist position of this research, the authors strived to uphold qualitative rigour and trustworthiness which is detailed in the strengths and limitations of the study. Moreover, the second and third authors’ respective qualifications in clinical psychology and physiotherapy allowed them to scrutinise biases in the findings related to health professional practice.

### Ethical considerations

This study was approved by the Human Research Ethics Committee at Western Sydney University (Approval Number: H13618). Pseudonyms were used to replace participants’ real names. Participants were informed of the option to withdraw during any point of the interview.

## Results

### Sample

The 21 participants’ ages ranged from 19 to 37 years old, with the mean age being 27.6 years. Except for one participant who identified as non-binary gender, all other 20 participants identified as a woman. One participant was an exchange student in Sydney when she sought help for vaginismus. Even though the participant returned to her home country, the eligibility criteria was extended to interview her about her help-seeking experiences in Australia only. The full demographic information of the sample of participants is presented in Table 1.

**Table 1:** Demographic information of participants

Demographic category	n	Demographic category	n
**Gender**		**Ethnicity**	
Woman	20	White, Caucasian, Anglo-Saxon/Australian, or European	15
Agender	1	Filipino	1
**Sexual orientation**		African	1
Heterosexual	17	Middle Eastern	1
Bisexual	2	Dutch	1
Pansexual	1	Indian	1
Greysexual	1	Vietnamese	1
**Religion**		**Vaginismus type**	
No religion	7	Primary	14
Anglican Christian	1	Secondary	3
Catholic	3	Primary and secondary (recurrent)	1
Lapsed Catholic	1	Unclear	3
Buddhist	1	**State where help was sought**	
Muslim	2	NSW	9
Atheist	3	SA	2
Agnostic	1	WA	1
Pagan witch	1	QLD	3
Other or missing	1	VIC	8
**Relationship status**		**Region type**	
Single	8	Metropolitan	21
In a relationship/married	13	Rural	3

### Thematic results

Four key themes emerged based on participants’ recommendations on how to improve help-seeking for vaginismus. These themes were: *Increase awareness of vaginismus*, *Dismantle myths about sex*, *Destigmatise vaginismus*, and *Empower people with vaginismus during medical consultations*. Subthemes emerged which represented the strategies participants recommended under each theme.

### Increase awareness of vaginismus

All 21 participants mentioned that there is a lack of knowledge that vaginismus is a diagnosable and treatable condition in the medical community and society. This delayed participants’ awareness that they had a problem which required seeking help. Participants advocated four main strategies, focused on *High school sex education*, *Health professional training*, *More research*, and to *Display vaginismus information at clinics*, to increase awareness of vaginismus to improve help-seeking.

#### High school sex education

Women advocated for female sexual dysfunctions, such as vaginismus, to be included in the high school sex education curriculum. They explained that early education on female sexual health can prevent delayed awareness of vaginismus. Crystal described the potential positive effect of high school sex education:

I feel like they could implicate maybe education within school. Because that’s when girls are figuring out their bodies. So to have awareness of not just like, you know, this is sex and stuff. They need to be like ‘all right, so if you have painful sex, which is common, this is what it could be. And if this happens, seek help’… So that way girls feel a lot more motivated to seek help about their issues and realise that they’re able to identify when there is a problem. It shouldn’t-, you shouldn’t have to be ages down, you know, adulthood when you figure out what’s actually happening.

As Crystal stated, participants shared that discussion of vaginismus symptoms in high school is needed as it is a context where young people are learning about their developing bodies. They contended that it would improve young people’s sexual health literacy. This, according to participants, can direct and empower young women to identify problems, such as difficult tampon insertion, and prompt help-seeking rather than ignoring it.

#### Health professional training

The second strategy participants recommended was for all healthcare professionals (HCPs) to undergo a course on female sexual health, which includes vaginismus, either during or after their formal training. Despite being physically examined, nine women were misdiagnosed with an anatomically narrow vaginal passage, lichen sclerosus, vulvodynia, endometriosis, and severe thrush. Women added that practicing HCPs should attend professional development to update their knowledge of female sexual dysfunctions. They discussed how more training for HCPs could improve the quality of healthcare that patients with vaginismus receive. Hope captured this sentiment as she explained:

If we can educate our medical professionals to just know that this [vaginismus] even exists. But also knowing how to deal with it. Even just the basics. Like obviously a GP, I don’t expect a cure from them in a 15 min appointment. But you know, if, if someone were to go to a GP and say,  ‘hey, I’m having painful sex’, I want that GP to have a base level of of education that allows them to ask a few pertinent questions and then say, ‘hey, I think you should maybe go and see a physiotherapist who specialises in pelvic pain.’ Just just something as simple as that I think could be incredibly beneficial.

Hope reflected several women’s comments that the HCPs they consulted were not aware of vaginismus and were unable to make sense of their symptoms. As Hope noted, it is not expected that all HCPs know how to diagnose and treat vaginismus. However, several participants stressed that training is needed to ensure that HCPs, especially first-line HCPs such as GPs and psychologists, have a general understanding of female sexual pain disorders. They contended that this would improve HCPs’ referrals of affected patients to appropriate services for treatment without delay.

#### More research

Participants advocated for more research to understand how women become aware of vaginismus to seek help for it. They called for research to better understand and promote the impact of the condition on women’s lives. Amy summarised women’s hope for more research on vaginismus:

It would be great to be able to have some more funding to be able to research, as you are, the impact that it has on people and create campaigns around, around these symptoms, particularly for young women who are perhaps just experiencing these symptoms for the first time.

Similar to Amy, several women explained that more research can be used to develop campaigns to raise awareness of the condition in ways that encourage women, and those who know women, with symptoms of vaginismus to seek medical help. They argued it can help people to better understand their bodies to not ignore their symptoms and empower women to seek help and treatment even after unsuccessful attempts.

#### Display vaginismus information at clinics

Women recommended the strategy of publicly displaying information that explains vaginismus and its symptoms as wall posters or desk pamphlets in general waiting areas of medical practices. They hoped this strategy would attract the attention of waiting patients and increase their awareness of vaginismus. Belinda elaborated on this strategy: “…in a normal clinic, you see like posters up about different like conditions in their office. If there was just one of those [on vaginismus], then women might actually read it and go, ‘oh yeah, that sounds like me’.” As Belinda highlighted, reading information on vaginismus in medical spaces can prompt women who may be experiencing the condition to seek help, and alert them to people to contact and places to gain help. Participants argued that displaying public information of vaginismus can instigate a snowball effect wherein it can prompt people, without the condition, to search vaginismus on Google and spread awareness of the condition through word of mouth in a relevant context.

### Dismantle myths about sex

Eighteen participants highlighted that there is a continued spread of misconceptions about sex. These misconceptions include sex being portrayed as spontaneous and painless in broader society and popular media. Participants recommended two strategies to *Revoke myth of painful sex* and *Question myth of spontaneous sex* to address misconceptions about sex for women in healthcare and broader society.

#### Revoke myth of painful sex

Participants called for HCPs and broader society to advocate revoking the myth that sex is supposed to be painful for women through education, medical training, and media. When sharing their experiences of painful sex, seven women had consulted a HCP who responded with unknowledgeable and dismissive advice to “take a bath,” “use more lubricant,” “drink alcohol,” or “relax”. Therefore, participants such as Amanda, called for:

More of an emphasis on like sex shouldn’t be painful. And you don’t need to put up with pain. And if you are having pain in sex, then you should go see a health professional who can help you to not have painful sex.

Amanda’s explanation reflected other women’s recommendation to counteract the perpetuation of the myth through magazines, friends, and media depictions of women losing their virginity with pain and bleeding. Participants advocated such a strategy to increase women’s ability to recognise painful, difficult and/or impossible vaginal penetration experienced when they first become sexually active as problematic. They posited that sort of strategy can reduce the incidence of those with primary vaginismus trivialising their symptoms and not immediately seeking help when they first experience sexual pain.

#### Question myth of spontaneous sex

Participants explained that the visual portrayal of sex and pleasure being spontaneous, effortless, and instant has led to female sexual dysfunctions such as vaginismus not being well-known. They called for the myth of spontaneous sex to be questioned to dismantle it in media representations which influences people’s beliefs and perceptions of sex. Chloe summarised that:

I guess in terms of just kind of general awareness, getting rid of this idea that, that all sex is amazing and passionate and going to just happen by the snap of our fingers every single time, get rid of that misconception. And also the misconception that sex is supposed to hurt for women at first. Taking away those kind of ideas, I think would help a kind of general awareness to know that that-, that’s not the case and other things can happen.

Chloe illuminated several women’s argument that questioning and critiquing the myth of spontaneous sex can help women in two main ways. It can uphold their sense of self-worth to understand that they are women even if penetration is difficult. Moreover, questioning the myth of spontaneous sex can help people to realise that it is very possible to not have spontaneous sex and to seek help for it.

### Destigmatise vaginismus

Seventeen participants discussed how female sexual health issues, especially regarding periods or the vagina, are viewed as ‘gross’ and taboo. They posited that this must be challenged to enable women to openly discuss their symptoms of vaginismus for help. This led to participants’ recommendations producing three strategies to *Promote discussion of vaginismus in healthcare*, *Change the name of vaginismus*, and *Public figure or media discussion.*

#### Promote discussion of vaginismus in healthcare

Participants exalted the strategy for people experiencing vaginismus and HCPs to become more comfortable discussing painful sex. Anna encapsulated this strategy when she explained:

One, I don’t think females feel comfortable talking about it [vaginismus] to begin with. And two, I don’t think practitioners really feel comfortable talking about it. I feel like it’s always been a bit taboo to openly speak about that ‘yes, sex is painful.’

As Anna explained, participants advocated this strategy to challenge the stigma regarding talking about vaginas even if it is not one’s area of expertise. Some called for HCPs to openly discuss vaginal problems and make appropriate referrals. They added it can help to promote women’s self-acceptance to reduce them feeling abnormal.

#### Change the name of vaginismus

Participants noted that the name ‘vaginismus’ sounds clinical and taboo. They suggested to change the name of the condition to something less likely to be stigmatised. Belle explained that:

You wouldn’t just like drop it in conversation because of the word. I wouldn’t be like ‘yeah like I have to go see a doctor because of vaginismus.’ But if I said ‘I, I have to go see a doctor because of my like Kitten’s disease’, people will just be like ‘OK.’ But when you say ‘vaginismus,’ they’re like ‘what’s wrong with her vagina?’

Other women, similar to Belle, noted that the word vaginismus evoked heavily medicalised jargon and imagery of hidden areas. They conveyed that changing the name of the condition to suit common usage would help to make it easier to talk about. This can reduce women’s feelings of the taboo to seek help according to participants.

#### Public figure or media discussion

Participants also strategised that the discussion of vaginismus by an influential figure or in media can help to destigmatise the condition. Olivia explained how “the only recent media depiction I’ve seen of vaginismus was on that Netflix show *Sex Education*. I don’t know if you’ve seen it, but one of the characters actually has vaginismus so that’s a start.” Olivia’s comment was reflected in several women’s statements that discussion of vaginismus by public figures or media can potentially reduce the taboo of vaginismus and encourage people to start talking about the condition in public. They noted that this could help women struggling with vaginismus to gain the support that they need. Participants pointed out that similar strategies have been employed to successfully begin to destigmatise polycystic ovarian syndrome and endometriosis. Participants added that such strategies can help women with vaginismus to not feel so alone and more normal to possibly discuss their issue openly to gain help.

### Empower people with vaginismus during medical consultations

Sixteen participants felt as though their HCP determined their course of action without their input, especially when engaging with private parts of their body. This enhanced the power imbalance between them and HCPs. Therefore, participants advocated two strategies for HCPs to *Provide treatment rationale* and *Verbal negotiation during physical examination and treatment* to empower people with vaginismus during medical consultations.

#### Provide treatment rationale

Participants highlighted that there were many instances when they did not understand or feel comfortable with treatment as it was not explained by their HCPs. They recommended that HCPs should give them information on why certain treatments or approaches are being used. Grace shared how it is necessary for:

… specialists being able to explain the treatment effectively and the efficacy of that treatment because it’s kind of strange in a way that you’re asking this person to take this dilator home and do these things and um yeah you need to kind of have that mind to say, ‘yep, I get why we’re doing this and I’m going to go home and do it’.

Several other participants, like Grace, proposed that patients should be informed about the uses, benefits and challenges of treatment. This could reduce the power imbalance between HCPs and patients according to participants. They suggested it can foster women’s trust that HCPs are not dictating what is best for them, but seeking to work with them.

#### Verbal negotiation during physical examination and treatment

Another strategy that participants advocated was for HCPs to let them know what they will be doing to women’s bodies and provide feedback as they do it. HCPs are accessing very private and personal parts of women’s bodies that often feel extreme pain. Therefore, participants stated that HCPs letting them know how long they will touch, press, or insert their fingers/hands on their bodies helps women to mentally prepare and endure the discomfort. Molly shared how:

Explaining what you’re doing and why you’re doing it. I think so many times I’ve been to health professionals that just do stuff to you… so, you know, sort of explaining that, why they need to know that and what they’re about to do and asking for permission, like she, she [HCP] asked every time she went to touch, like a different area of my abdomen or my back or whatever, she she would tell me what she was doing. She’s like ‘is it okay if I touch your hip here? I just want to assess X, Y, Z’.

Molly articulated how alerting women about touch before doing so promotes informed consent and fosters a therapeutic alliance between professionals and patients. Consequently, women stated such negotiation would make them feel like they are being respected enough to be constantly informed as agentic beings rather than clinical objects to be acted on.

## Discussion

The participants in this study identified issues in the healthcare system and society when help-seeking for their vaginismus. The feminist constructivist approach used to centre participants’ voices recognised them as producing insider knowledge to improve the issues that they identified. All participants advocated change through their recommendations to *Increase awareness of vaginismus*, *Dismantle myths about sex*, *Destigmatise vaginismus*, and *Empower people with vaginismus during medical consultations.* These recommendations have implications for practice and reflect the need to incite a culture of change in healthcare and broader society [36, 37].

All participants unanimously reflected on the societal lack of awareness of vaginismus, which led to many not realising that vaginismus was a diagnosable condition. As a result, they were unaware of the need to voice their problems and seek healthcare, which subsequently caused their delay to begin help-seeking [14]. If one never gains the awareness to seek help for their symptoms, they lack the necessary sexual health literacy and self-determination to improve their health [38]. Instead, their sexually uninformed sense of self would continue to not seek help and vice versa, perpetuating their lack of sexual health literacy and inaction, like a feedback loop. The strategies that participants advocated to raise societal awareness for vaginismus calls for a systemic culture change.

This has implications for education developers to change their practice, design, and delivery of sexual health curriculums to appropriately incorporate discussion of painful sex in high school and tertiary qualifications for healthcare professionals (HCPs). This will increase social awareness of vaginismus among the public if they gain general knowledge about it in their adolescence through high school education to recognise the condition as a problem to seek help. Moreover, it can improve HCPs’ knowledge of the condition to improve their practice to display information on vaginismus at clinics like participants suggested, and offer direct support or referrals for women to better understand their bodies [39]. This can then influence cultural change at the individual level for women to feel less shame, embarrassment, and comfort to discuss the condition to gain appropriate help [36, 40].

The current societal ignorance of vaginismus is a form of marginalisation and silencing of women’s voices to advocate and make sense of their symptoms to seek help [5]. This is not necessarily deliberate. However, unlike the capitalist drive to publicise erectile dysfunction through mainstream media to market Pfizer’s Viagra (sildenafil), there is no equivalent pharmaceutical or political agenda to raise awareness for female sexual dysfunctions such as vaginismus. This renders a culture where myths of spontaneous sex and painful sex for women being normal perpetuate, especially the latter in healthcare spaces as participants revealed [41, 42]. Interventions focused on myth busting or transformative learning to address problematic beliefs and unconscious biases should be implemented at the organisational level in healthcare practice. Specifically, such interventions should be included in professional development courses or events, such as conferences, training seminars, or orientation coursework organised by practice managers for newly hired HCPs joining a clinic. This can drive cultural change to make people with vaginismus to feel safer with, and better understood by, HCPs [40].

The strategies that participants advocated to train HCPs to understand vaginismus and inform women about the need, benefits and challenges of treatment can be incorporated at a systemic level in healthcare to improve professional practice [43, 44]. Such strategies have practice implications to reduce the number of HCPs that patients need to consult to finally gain an appropriate diagnosis and options for interventions that are most suited to their presentation of vaginismus [39]. This can facilitate a culture change to destabilise the power imbalance between patients and professionals and foster a better therapeutic alliance to increase women’s ability to access information, diagnosis, treatment, and health services [44]. It can foster positive help-seeking experiences which allow women to feel supported by the healthcare system to be receptive of HCPs’ therapeutic approach.

The implementation of the strategies that participants advocated can help to incite a culture change in society and healthcare that can lead to more focus on the empirical study of vaginismus symptoms within the organisational levels of universities and companies [44]. Further study of vaginismus is necessary given the current lack of understanding into the pathophysiology, cause, and consequences of vaginismus, which has led to inconsistency in defining the condition. While the DSM-5 conflates vaginismus and dyspareunia into genito-pelvic pain/penetration disorder (GPPPD) [45], the International Statistical Classification of Diseases and Related Health Problems (ICD)-11 reconceptualised vaginismus to sexual pain-penetration disorder and separates it from dyspareunia and vulvodynia [46, 47]. Empirically clarifying the symptoms and meaning of vaginismus is an essential step for practice. Only then is it possible to be closer to gain international consistency in the diagnostic criteria for vaginismus. Such international diagnostic consistency has practice implications to reduce the incidence of misdiagnosis and improper differential diagnosis which nine women experienced in this study [48]. This can help to clarify the understanding of vaginismus among HCPs that participants identified as an issue to help them to receive more effective healthcare support for vaginismus.

Patients’ voices continue to be unheard in the healthcare system [49]. Despite the recent shift to patient-centred care, changes that are implemented in the healthcare system tend to centre on HCPs’ expert opinion. This causes an indirect paternalism in healthcare, especially in the treatment of female sexual health conditions such as vaginismus. Participants’ recommended strategies in this study focused on HCPs offering more information on the rationale of treatments and physical examinations, which includes verbal negotiation to uphold informed consent. This highlights that women with vaginismus seek to participate more in the decision-making process of their healthcare management [50]. Since patient-centred care is a model of primary care that focuses on teamwork to improve patients’ help-seeking experiences [50], it is essential that HCPs’ practice gives patients with vaginismus the space and time to advocate their needs and concerns. This has implications for HCPs to segue into actively collaborating with patients to ensure their satisfaction, which evidence shows to improve treatment adherence and outcomes [51].

Participants’ recommendations in this study also focused on increasing awareness and decreasing the stigma of vaginismus in broader society. They praised the positive media representation of vaginismus and its treatment with vaginal trainers in the Netflix series *Sex Education*. Participants called for similar advocacy of vaginismus by popular media and influential figures. Advocacy focused HCPs and researchers could potentially seek partnerships with influential figures and media as part of their practice. This can help to maximise on the benefits of health advocacy by influential media and figures to cause positive social change and diminish stigmas towards vaginismus in ways that are not co-opted to perpetuate myths about women’s sexual health [52]. Such outcomes will enable women to receive correct health information about vaginismus. It also highlights that strategies to improve the awareness and stigma of vaginismus should be intertwined between health and public spheres.

### Strengths and limitations of the study

To our knowledge, this is the first study to explicitly ask those who have been diagnosed with vaginismus for their recommendations to improve healthcare and help-seeking for the condition. These findings can help to inform health professionals, researchers, or policy makers’ plans to implement change to improve healthcare practice for vaginismus. To uphold qualitative rigour and trustworthiness of findings, the researchers and participants ‘member checking’ the data established credibility [53]. For transferability, descriptions of the research protocol, including data collection strategies, thematic construction, and feminist approach, are provided to allow readers to assess whether the findings are applicable to other contexts [54]. To uphold dependability, the methodological design has been detailed for study replication [54]. Constructive feedback gained through internal and field testing of the interview guide sought to reduce bias [27]. Participants’ quotes are presented in the results to demonstrate that the findings are data driven.

Even though the study sought to centre and advocate participants’ voices through a feminist perspective, the researchers’ bias may have informed the categorisation of participants’ responses into the key recommendations. The sample had a low number of participants who were gender or sexually diverse. Therefore, the participants’ recommendations presented in this study tend to advocate cis-gendered and heterosexual women’s perspectives.

## Conclusion

This study used a feminist approach to collate the marginalised perspectives of people, who have been diagnosed with vaginismus, to advocate their concerns and strategies to improve help-seeking for vaginismus. Existing recommendations for help-seeking for vaginismus in the literature has a clinical focus on symptoms, treatment, and tend to be from health professionals’ and researchers’ perspectives. This study found that recommendations from patients, who have been diagnosed with vaginismus, focus on increasing awareness and decreasing stigma of the condition in both healthcare systems and broader society. To avoid paternalism in implementing change, health professionals, researchers, and policy makers need to seek the perspectives and recommendations of those with vaginismus and collaborate with them to generate meaningful change. This can promote women’s participation in their own healthcare for vaginismus. It will lead to changes in approaches to help-seeking for vaginismus that better align the expectations and perceptions of healthcare professionals and their patients when treating the condition. Ultimately, these changes are required before people with vaginismus can seek help more effectively and see meaningful improvements in the therapeutic alliance and treatment outcomes.

### Electronic supplementary material

Below is the link to the electronic supplementary material.

Supplementary Material 1

Supplementary Material 2

## Data Availability

The dataset generated and/or analysed during the current study are not publicly available due to reasons of sensitivity and participant privacy, and are only available from the corresponding author upon reasonable request. Data is located in restricted access data storage at 10.26183/sw56-z237.

## Abbreviations

DSM: Diagnostic and Statistical Manual
GPPPD: Genito–pelvic pain/penetration disorder
HCP: Healthcare professional
HCPs: Healthcare professionals
ICD: International Statistical Classification of Diseases and Related Health Problems
SRQR: Standards for Reporting Qualitative Research
