# Supplementary material for: Patients’ recommendations to improve help-seeking for vaginismus: a qualitative study
Source: BMC Womens Health. 2024 Mar 30;24:203. doi: 10.1186/s12905-024-03026-x (PMC10981325; doi:10.1186/s12905-024-03026-x)
Supplement: Supplementary file 1 — Supplementary Material 1 [file 12905_2024_3026_MOESM1_ESM.docx]

Supplementary Table 1. Demographic interview questions

| How old are you? |
| --- |
| How old were you when you were diagnosed with vaginismus? |
| Have you been cured, or do you continue to have vaginismus? |
| What state and city did you seek help for vaginismus in? |
| What is your postcode? Was that the same postcode you had when you sought help for vaginismus? |
| What is your sexual orientation? |
| What is your relationship status? |
| What gender do you identify as? |
| What is your ethnicity? |
| What are your religious beliefs, if any? |
| What is your highest level of education? |
| Are you employed? If you are, what do you work as? |
| Do you work casually, part-time, or full-time? |
| I am asking this next demographic question because we’re interested to see if there is a correlation between women’s income and their ability to access and afford specialist health services for vaginismus. So, if you don’t mind me asking, is your income below $50k, between $50k–100k, between $100k–$200k, or above $200k? |
| What would you describe your socio-economic status as? |
