# Supplementary material for: Patients’ recommendations to improve help-seeking for vaginismus: a qualitative study
Source: BMC Womens Health. 2024 Mar 30;24:203. doi: 10.1186/s12905-024-03026-x (PMC10981325; doi:10.1186/s12905-024-03026-x)
Supplement: Supplementary file 2 — Supplementary Material 2 [file 12905_2024_3026_MOESM2_ESM.docx]

Supplementary Table 2. Final semi-structured interview guide

| **Overarching Question** | **Potential probes where relevant or appropriate** |
| --- | --- |
| ***I. Tell me about your journey to get help for vaginismus*** | How long did it take you to realise that you needed to seek help after noticing symptoms? |
|  | Who did you first talk to about your symptoms? |
|  | What motivated you to seek help for vaginismus in the first place? |
|  | How did you gain a diagnosis? |
|  | How long did it take to receive a diagnosis? |
|  | How long have you sought help for vaginismus? |
|  | Did you hear about vaginismus before you realised that you had symptoms? |
|  | Do you think vaginismus is a well-known condition and why? |
|  | What treatment/s do/did you receive? |
|  | How did you find the costs to get help? |
|  | ^¥^Research in Australia shows that people from non-Anglo backgrounds face barriers to access the healthcare that they need. Do you think your ethnic or racial background had any influence on getting help for vaginismus? |
|  | ^¥^Do you think your cultural and/or religious upbringing had any influence on how you sought help? |
|  | ^¥^How has having… (insert pelvic pain co-morbidity) affected your journey to get help for vaginismus? |
| ***II. What were the best and worst parts of your journey to seek help?*** | What did you find helpful about the healthcare system? |
|  | What did you find unhelpful about the healthcare system? |
|  | What treatment/s did you find helpful? |
|  | What treatment/s did you find unhelpful? |
|  | How well do you feel health professionals support you for vaginismus? |
|  | How does the way health professionals support you make you feel about treatment for vaginismus? |
|  | Have you experienced any barriers to getting adequate treatment? |
|  | How could we reduce the barriers you’ve faced? |
| ***III. What has your experience of treatment for vaginismus been like?*** | How do/did you feel having to undergo specific treatments for vaginismus such as… (insert treatments that participant named)? |
|  | How does/did undergoing treatment for vaginismus make you feel about continuing treatment? |
|  | How does the information that health professionals provide make you feel about treatment? |
|  | Did/Do you feel like the ability to get better from vaginismus was/is in your control and why? |
|  | Did/Do you feel like the ability to treat vaginismus depended/depends on any other external factors? |
|  | Do you think that you will be cured if you pursue treatment? Why? |
|  | ^¥^If you have completed treatment, do you feel cured? Why or why not? |
| ***IV. What impact does/did seeking help for vaginismus have on your sense of self?*** | What made you persevere and seek help even when you faced barriers? |
|  | How does/did undergoing treatment for vaginismus make you feel about yourself? |
|  | What impact has the journey to seek help had on your sense of womanhood?   - ^¥^Do you feel identifying as non-binary gender has impacted your journey to seek help and your sense of womanhood? - ^¥^Do you feel that not being heterosexual has impacted your journey to seek help and your sense of womanhood? |
|  | How has seeking help for vaginismus made you feel about yourself in relation to other women who do not experience the problems of vaginismus? |
|  | What impact has this journey to find help had on your sense of being a sexual being?   - ^¥^Do you feel that not being heterosexual has played any part in seeking help and its impact on your sense of self as a sexual being? |
|  | Has your journey to seek help had any impact on your mental and emotional wellbeing? |
|  | What are your recommendations to improve the help-seeking process for vaginismus? |
|  | Would you like to share anything else about your experiences with the healthcare system, health professionals, or anything else about seeking help for vaginismus? |
|  | What made you want to participate in this study? |
| ^¥^ indicates conditional questions that were only asked to applicable participants. | |
